# Filial cannibalism influences the link between gonadal development and antioxidant function in a mouthbrooding cichlid fish

**DOI:** 10.17912/micropub.biology.001352

**Published:** 2024-11-20

**Authors:** Hailey A Hartman, Howard A Mitchell, Peter D Dijkstra

**Affiliations:** 1 Biology, Central Michigan University, Department of Biology, Mt. Pleasant, MI, USA

## Abstract

In females of the mouthbrooding cichlid fish *Astatotilapia burtoni* , we recently found a positive relationship between liver antioxidant function and filial cannibalism. Here, we manipulated the level of fry consumption in *A. burtoni* females to assess how the level of fry consumption affects liver antioxidant function. Feeding treatment did not affect liver antioxidant function, but feeding treatment significantly influenced the relationship between gonadal development and antioxidant function. Future studies should isolate the effects of gonadal development and fry consumption on antioxidant function.

**Figure 1. The effect of feeding fry on liver antioxidant function and gonadal development in female cichlid fish:**
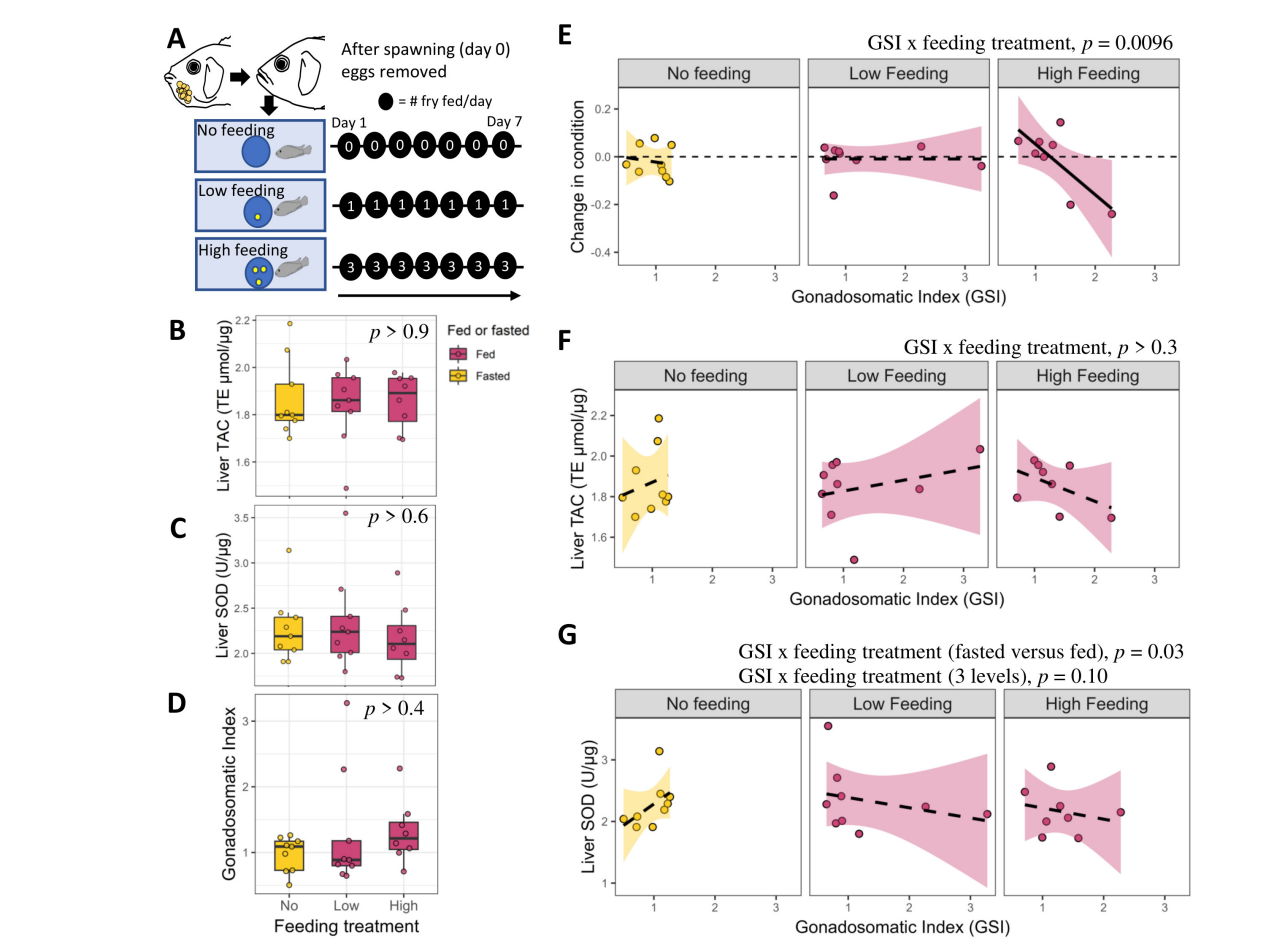
**Figure 1. A.** Experimental design. Females were either not fed or fed 1 fry per day (low feeding) or 3 fry per day (high feeding treatment) for 7 days. Tissue was collected on day 8. **B.** Liver total antioxidant capacity (TAC) by feeding treatment. **C.** Superoxide dismutase (SOD) activity by feeding treatment. **D.** Gonadosomatic Index (GSI) by feeding treatment. Boxes enclose 25th to 75th percentiles. Error bars enclose data range, excluding outliers. **E.** Change in body condition as a function of gonadosomatic index plotted for each feeding treatment. The change in body condition was calculated as the body condition on day 8 (at tissue collection) minus that on day 0 (after egg removal) divided by body condition on day 0. Dashed lines represent nonsignificant relationships and the solid line represents a significant relationship. **F.** Liver total antioxidant capacity (TAC) as a function of gonadosomatic index plotted for each feeding treatment. **G.** Superoxide dismutase (SOD) activity as a function of gonadosomatic index plotted for each feeding treatment.

## Description

The high energetic demands of reproduction may constrain future fitness and longevity leading to life history trade-offs (Daan et al., 1990; Stearns, 1992; Williams, 1966) . Consequently, parents may reduce investment in current reproduction (e.g. reduced parental care) to promote somatic maintenance and future reproductive success. Oxidative stress, which occurs when the production of reactive oxygen species (ROS) overwhelms the antioxidant system, has been suggested as a potential mediator of life-history trade-offs (Selman et al., 2012; Speakman & Garratt, 2014) . The high metabolic cost of parental care increases the production of ROS, potentially causing oxidative stress (Monaghan et al., 2009) . However, many studies have found no effect or even reduced oxidative damage in parents that were forced to increase investment in parental care (Garratt et al., 2013; Ołdakowski et al., 2015). This is due to animals’ ability to manage or minimize potential oxidative challenges of reproduction through behavioral or physiological adjustments (Blount et al., 2016; Hood et al., 2018) . Identifying potential strategies that animals use to cope with oxidative challenges may provide important insights into the role of oxidative stress in mediating life-history trade-offs (Costantini, 2019) .

In the cichlid fish *Astatotilapia burtoni* , females exhibit maternal care in the form of mouthbrooding (Maruska et al., 2020; Sawecki et al., 2019) . During mouthbrooding, females hold their fertilized eggs and developing embryos in the buccal cavity for approximately two weeks (Renn et al., 2009) . During this time, the female is unable to consume food and consequently experiences obligatory starvation (Faber-Hammond & Renn, 2023; Ray et al., 2023) . The lack of feeding may prevent the female from replacing energy reserves and dietary antioxidants, which may compromise the mother’s ability to maintain redox homeostasis (Di Simplicio et al., 1997; Grattagliano et al., 2000) . However, in a previous study, we found that the number of embryos varied significantly across stages of mouthbrooding, suggesting that mothers may ingest some of their offspring during mouthbrooding (Sawecki & Dijkstra, 2022) , as has been reported for other cichlid fish (Mrowka, 1987) . We also found that the level of filial cannibalism was positively related to liver antioxidant function. If increased antioxidant function helps mothers to maintain redox balance under these conditions, the observed cannibalism suggests that mothers are sacrificing offspring for increased future reproductive success (Sawecki & Dijkstra, 2022) . However, in this previous study we took advantage of natural variation in the rate of filial cannibalism and hence we do not know the directionality of the relationship between filial cannibalism and liver antioxidant function in the mothers. In this study, we manipulated fry consumption in *A. burtoni* females to assess how the rate of fry consumption affects antioxidant function in the liver in terms of total antioxidant capacity (TAC) and the enzymatic antioxidant superoxide dismutase (SOD). Our approach was to collect females who had recently spawned, remove their eggs, and then randomly assign them to three treatment groups: the fasting treatment (7 days without food), low fed treatment (one fry per day), and high fed treatment (three fry per day) ( **Fig. 1A )** . We then collected livers on day 8 to quantify liver antioxidant function in these females.

We found that liver antioxidant function in females was not affected by feeding treatment relative to both total antioxidant capacity ( **Fig. 1B** , LM, effect of treatment: F _2, 23_ = 0.067, p = 0.94) and SOD ( **Fig. 1C** , LM, effect of treatment: F _2, 23_ = 0.3628, p = 0.70). There were also no significant differences in liver antioxidant function between fed and starved females (p values > 0.76). These findings are contrary to the expectation that fry consumption boosts antioxidant function. However, we found that feeding fry promoted gonadal development in some females. Although feeding did not significantly increase final gonadosomatic index (GSI) (LM, effect of treatment: F _2, 23_ = 0.778, p = 0.47), the range in GSI values is wider in fed females compared to starved females ( **Fig. 1D** ) suggesting that feeding promoted the maturation of eggs. Previous studies have shown that gonadal development and egg production are metabolically costly (Salmon et al., 2001; Vézina & Williams, 2005) . Therefore, we examined the link between final GSI and change in body condition over the 7-day experiment defined as the percentage change in body condition at the time of tissue sampling relative to the body condition of the female at the onset of the feeding experiment (immediately after egg removal). We found that final GSI was linked to a change in body condition during the 7-day experiment in a treatment-dependent fashion (LM, GSI x treatment, -0.012 ± 0.004, t = -2.836, p = 0.0096, **Fig. 1E** ). Specifically, there was a significant negative relationship between change in body condition and final GSI in the high feeding treatment (LM, final GSI, -0.211 ± 0.078, t = -2.702, p = 0.036) but final GSI did not influence change in body condition in the other treatments (p values > 0.68). These results indicate that feeding treatment might influence both gonadal development, which in turn might modulate antioxidant function. To test this idea, we examined how final GSI and feeding treatment is linked to liver antioxidant function. Final GSI and feeding treatment did not influence liver total antioxidant capacity ( **Fig. 1F** ). However, liver SOD was influenced by final GSI but this effect tended to differ across feeding treatments ( **Fig. 1G** ). This interaction effect was marginally nonsignificant (LM, GSI x treatment: F _2, 19_ = 2.552, p = 0.10; Body weight: F _1, 19_ = 5.2686, p = 0.03). However, when both feeding treatments were combined into one category the interaction between final GSI and feeding treatment (fed versus fasted females) was significant (LM, final GSI x fed v. fasted treatment, 1.215 ± 0.526, t = 2.312, p = 0.03; Body weight: 0.40 ± 0.14, t = 2.848, p = 0.0096). This interaction effect was driven by marginally nonsignificant negative and positive relationships liver SOD and GSI within the starved and the fed females, respectively (fed: -0.278 ± 0.152, t = -1.827, p=0.09; starved: 0.999 ± 0.424, t = 2.356, p = 0.057). These links between antioxidant function and gonadal development are consistent with the previously described oxidative cost of egg production and maturation in a range of organisms (Bertrand et al., 2006; Wang et al., 2001) including our species (Sawecki & Dijkstra, 2022) .

Filial cannibalism enables parents to recoup some of the lost energy they put into their offspring (Fischer & O’Connell, 2017; Manica, 2002) and it seems likely it would also boost antioxidant function since food consumption is known to promote the antioxidant capacity in many organisms (Chen et al., 2022; Copple et al., 2008) . Here we experimentally tested whether fry consumption boosts liver antioxidant function. However, our findings were confounded by feeding treatment influencing gonadal development, with the latter likely modulating antioxidant function. Future studies should manipulate fry consumption in mouthbrooding females who are still mouthbrooding, which halts gonadal development. It may be difficult, though, to control fry consumption when females are holding the brood in their mouth. Alternatively, arresting gonadal development using either pharmacology or short photoperiod (Biswas et al., 2005) might be a promising avenue to isolate the effect of filial cannibalism on liver antioxidant function.

In the current study, we revealed complex links between feeding regime (fry consumption), gonadal development, and antioxidant function. Our data supports the notion that cannibalism influences the potential role of oxidative stress in mediating the trade-off between investment in current and future reproductive

## Methods

*Experimental Animals and Housing*

*Astatotilapia burtoni* used in this experiment were from a laboratory-bred population that was derived from wild-caught fish from Lake Tanganyika, Africa. Fish were housed in 100-liter tanks with a re-circulating life support system at 28 °C on a 12:12 light: dark cycle with a 10 min dawn and dusk periods to mimic their natural habitat. All tanks contained gravel to facilitate substrate digging behavior with shards of terra cotta flowerpots to serve as territorial habitats. Fish were fed daily with flake food, unless indicated otherwise. All procedures are approved by the Institutional Animal Care and Use Committee (IACUC, protocols #18-10).

*Experimental Procedure*

Females were housed in seven 100-liter breeding tanks each containing 18-21 females and 2-3 males with halved terracotta flowerpots to promote breeding. We observed fish daily in the morning to identify females that had just spawned and started mouthbrooding. Once a female started mouthbrooding, we removed the eggs, and transferred each female to a 100-liter aquarium. The number of eggs of each experimental female were then counted. Experimental females were housed individually with a halved terracotta flowerpot in the middle of each experimental tank for shelter. Tanks were separated by brown paper but we ensured that each isolated female had visual access to another female in one neighboring tank by folding one of the top corners of the brown paper. This formed approximately a 3cm triangle that allowed females to see one neighboring female to avoid social deprivation while not inducing excessive territorial behavior (Dijkstra et al., 2007; Funnell et al., 2022) . The individually housed females were randomly assigned to three treatment groups: the fasting (no feeding) treatment (n=9), low fed treatment (n = 9), and high fed treatment (n = 9). For the fasting treatment, the females were not fed for seven days. For the low fed treatment, the females were fed one fry per day for 7 days (females were transferred on day 0, first feeding took place on day 1). For the high fed treatment, the females were fed three fry per day for 7 days which was below the maximum number of 25 fry females were able to consume in our previous study. Feeding took place in the morning. To feed the females the fry, a 5cm diameter petri dish was placed on the bottom of the tank near a flowerpot (at the center of the tank) then developing fry with the yolk sac present were inserted on the petri dish via pipette (in the fasting treatment, females were subjected to the same procedure but no fry was given). On the morning of day 8 (the day after the final feeding or sham treatment), females were netted, and their weight and standard length were measured prior to decapitation. The brain was removed for a different project. We then dissected and weighted the gonads. The livers were dissected and flash frozen in liquid nitrogen. Frozen liver samples were stored at -80 ^o^ C until further analysis. One female in the high fed treatment was excluded from the analysis because she was still mouthbrooding at the end of the experiment.

*Quantifying Antioxidant Capacity and Oxidative Stress*

We followed established procedures (Fialkowski et al., 2021) to evaluate total antioxidant capacity (TAC) and superoxide dismutase (SOD) activity in the liver, which were the same markers of antioxidant function that were assessed in our previous study (Sawecki & Dijkstra, 2022) . TAC and the amount of SOD content were reported per μg of protein.

*Statistical Analysis*

The statistical package R v3.6.1 was used for all statistical analyses of the data. Gonadosomatic index (GSI) was calculated as (gonad weight/total body weight)*100. We calculated body condition as weight/(length) ^3^ × 100 (Bolger & Connolly, 1989) . The change in body condition was calculated as the body condition on day 8 (at tissue collection) minus that on day 0 (after egg removal) divided by the female’s body condition on day 0. We used linear models (LMs) to test whether variation in our markers of antioxidant function or change in body condition could be explained by feeding treatment and/or variation in gonadal development using gonadosomatic index at the moment of tissue collection. We used Tukey’s multiple comparison post hoc tests for pairwise comparisons between the three treatment groups. In our models, we also considered the effect of the number of eggs produced (before the start of the feeding experiment) and body weight (at the beginning of the experiment after egg removal) as potential covariates. These covariates were only retained when they had a significant effect. A significance level (α) of 0.05 was used for all tests. We report mean ± SE for our model estimates.
